# Quantifying on-water performance in rowing: A perspective on current challenges and future directions

**DOI:** 10.3389/fspor.2023.1101654

**Published:** 2023-03-16

**Authors:** Martyn J. Binnie, Daniel Astridge, Sophie P. Watts, Paul S. R. Goods, Anthony J. Rice, Peter Peeling

**Affiliations:** ^1^ Department of Performance Sciences, Western Australian Institute of Sport, Perth, WA, Australia; ^2^ School of Human Sciences (Exercise and Sport Science), The University of Western Australia, Perth, WA, Australia; ^3^ Murdoch Applied Sports Science Laboratory, Murdoch University, Perth, WA, Australia; ^4^ Centre for Healthy Ageing, Health Futures Institute, Murdoch University, Perth, WA, Australia; ^5^ Sports Science, Rowing Australia, Canberra, ACT, Australia

**Keywords:** power, velocity, environment, competition, training

## Abstract

Winning times at benchmark international rowing competitions (Olympic Games and World Championships) are known to vary greatly between venues, based on environmental conditions and the strength of the field. Further variability in boat speed for any given effort is found in the training environment, with less controlled conditions (i.e., water flow, non-buoyed courses), fewer world class competitors, and the implementation of non-race specific effort distances and intensities. This combination of external factors makes it difficult for coaches and practitioners to contextualise the performance underpinning boat speed or race results on any given day. Currently, a variety of approaches are referenced in the literature and used in practice to quantify this underpinning performance time or boat speed, however, no clear consensus exists. The use of relative performance (i.e., time compared to other competitors), accounting for influence of the weather (i.e., wind and water temperature), and the novel application of instrumented boats (with power instrumentation) have been suggested as potential methods to improve our understanding of on-water rowing speeds. Accordingly, this perspective article will discuss some of these approaches from recent literature, whilst also sharing experience from current practice in the elite environment, to further stimulate discussion and help guide future research.

## Introduction

International rowing regattas are currently contested over 14 events at the Olympic Games, with a further 6 lightweight events included at the annual World Championships (Table 1). Akin with all racing sports, the result that ultimately matters in rowing is the outcome relative to other competitors at the benchmark events (placing). However, understanding the performance (race completion time or boat speed) that underpins this outcome is crucial towards informing a myriad of factors such as athlete selection, monitoring of progression, and successful preparation for and execution of a world class result. Notwithstanding, outside of benchmark events, it must also be considered that all training and preparation usually occurs in the home environment, which is often associated with less controlled conditions including flowing rivers and non-buoyed courses. Therefore, importance must also be placed on approaches that help with the interpretation of performance differences shown between varying training and competition environments.

**Table 1:** World's best times (WBT; current to end of December 2022) across Olympic games (OG) and world championships (WC) boat classes for both men and women, including date and location they were set. Presented in comparison to winning events times at 2021 Olympic games (Sea Forest Waterway/Tokyo, Japan) and 2022 World Championships (Racice, Czech Republic) across each of these boat classes (source, https://worldrowing.com/).

Event Category	Boat Class	WBT (mm:ss.00)	Date	Location	Winning Times 2021 OG (mm:ss.00)	Margin to WBT (%)	Winning Times 2022 WC (mm:ss.00)	Margin to WBT (%)	Margin Between Winning Times OG vs. WC (%)
OG	M1x	06:30.74	18/06/2017	Malta/Poznan, Poland	06:40.45	2.5%	06:48.67	4.6%	2.1%
	M2x	05:59.72	29/08/2014	Bosbaan/Amsterdam, Netherlands	06:00.33	0.2%	06:09.34	2.7%	2.5%
	LM2x	06:05.33	28/07/2021	Sea Forest Waterway/Tokyo, Japan	06:06.43	0.3%	06:16.46	3.0%	2.7%
	M2-	06:08.50	28/07/2012	Dorney Lake Eton/London, Great Britain	06:15.29	1.8%	06:28.06	5.3%	3.5%
	M4x	05:32.03	28/07/2021	Sea Forest Waterway/Tokyo, Japan	05:32.03	0.0%	05:40.08	2.4%	2.4%
	M4-	05:37.86	25/05/2012	Rotsee/Lucerne, Switzerland	05:42.76	1.5%	05:48.29	3.1%	1.6%
	M8+	05:18.68	18/06/2017	Malta/Poznan, Poland	05:24.64	1.9%	05:24.41	1.8%	−0.1%
WC	LM1x	06:41.03	9/09/2018	Lake/Plovdiv, Bulgaria			07:03.40	5.6%	
	LM2-	06:22.91	30/08/2014	Bosbaan/Amsterdam, Netherlands			06:47.69	6.5%	
	LM4x	05:42.75	30/08/2014	Bosbaan/Amsterdam, Netherlands			05:56.66	4.1%	
OG	W1x	07:07.71	21/09/2002	Guadalquivir/Seville, Spain	07:13.97	1.5%	07:31.66	5.6%	4.1%
	W2x	06:37.31	29/08/2014	Bosbaan/Amsterdam, Netherlands	06:41.03	0.9%	06:47.77	2.6%	1.7%
	LW2x	06:41.36	28/07/2021	Sea Forest Waterway/Tokyo, Japan	06:47.54	1.5%	06:54.78	3.3%	1.8%
	W2-	06:47.41	28/07/2021	Sea Forest Waterway/Tokyo, Japan	06:50.19	0.7%	07:03.76	4.0%	3.3%
	W4x	06:05.13	28/07/2021	Sea Forest Waterway/Tokyo, Japan	06:05.13	0.0%	06:17.49	3.4%	3.4%
	W4-	06:14.36	30/08/2014	Bosbaan/Amsterdam, Netherlands	06:15.37	0.3%	06:26.40	3.2%	2.9%
	W8+	05:52.99	28/07/2021	Sea Forest Waterway/Tokyo, Japan	05:59.13	1.7%	06:01.14	2.3%	0.6%
WC	LW1x	07:23.36	9/07/2022	Rotsee/Lucerne, Switzerland			07:42.59	4.3%	
	LW2-	07:18.32	6/09/1997	Lac/Aiguebelette, France			07:38.19	4.5%	
	LW4x	06:15.95	30/08/2014	Bosbaan/Amsterdam, Netherlands			06:38.14	5.9%	
**Average Margin (%; OG boat classes only)**						**1.1%**		**3.4%**	**2.3%**
**Range (%)**						**0.0%–2.5%**		**1.8%–5.6%**	**−0.1%–4.1%**

M, Men's; W, Women's; L, lightweight; 1x, single sculls; 2x, double sculls; 2-, *p*air; 4x, quadruple sculls; 4-, four; 8+, eight.

In more closed environment racing sports such as swimming and track cycling, we can look to the improvement of athletic attributes, training methods, technology and/or tactics to predominantly explain shifts in performance, as reflected by World's best times (WBT). However, in on-water racing sports such as rowing, the added complexity of a dynamic environment (i.e., wind, water temperature) makes a given performance difficult to quantify. Interestingly, we can look to the most recent Olympic Games (Tokyo 2021) as an example, with new Olympic best times being set in 12/14 events, with 6 of these times also new WBTs (Table 1). Of note, 10/12 of these new Olympic best times, including all the 6 WBTs were set on the same day (28th July 2021). While it is tempting to argue that this may point to a rational improvement in athlete performance at the most recent peak event, it is hard to ignore the potential contributing factors of a favourable course and weather conditions, especially when we consider the slower relative times across almost every event (∼2.3% on average) at the recent 2022 World Championships in the Czech Republic (Table 1). In fact, historical weather information (accessed *via* https://www.timeanddate.com/weather/) for the specific dates and locations of these events highlight a substantial difference in weather conditions that may, in part, explain the difference in performance times observed (Tokyo, 23–30 July, 2021—approximately 31°C and 5.6 m/s average wind speed, Czech Republic, 18–25 September, 2022–15°C and 2.0 m/s average wind speed). To further support this argument for event (venue and time of year) specific influence, 12/20 rowing event WBTs are now shared between just 2 regatta courses (Sea Forest Waterway/Tokyo, Japan and Bosbaan/Amsterdam, Netherlands; Table 1). While athlete, training and technology improvement is likely a factor explaining some of this improved performance standard, the underlying contributions, especially the effect of environment, remain largely unknown.

It should be acknowledged that the variability of winning race times in rowing is not a new discovery (1, 2), however, there remains no clear consensus as to the best way to account for this variation to contextualise race results or to scale any given training effort. In the hope to further stimulate the collective knowledge in the sport, we will discuss some of the available information coupled with experience from daily practice in an elite training environment. For instance, it is suggested that consideration must be given to a range of factors including the strength of competition [including the event stage (i.e., heat vs. final) which could influence athlete pacing and effort], speed of the environment (race venue and weather conditions), and the specific boat class when trying to account for factors that may influence performance (2). With this in mind, we suggest that an improved understanding from power instrumentation could further assist in the assessment of both individual and crew performances independent of raw performance times or placings alone (3, 4). Finally, given the amount of time spent in the training environment, we must consider the transfer and relevance of performances between preparation and competition environments. New knowledge in this area will assist coaches and practitioners towards a more informed understanding of the underlying performance standard required to achieve a desired race outcome.

In this perspective article, we will discuss current challenges and recent advancements in assessing performance and progression within the on-water arena for rowing, with a goal to help inform best practice considerations and highlight future research needs.

## Relative performance

The use of relative times to index performance is widely reported as common practice in rowing (5–7). Specifically, WBT for each boat class (Table 1) are used to provide a fixed benchmark from which to index a percentage of relative speed, often termed a “prognostic” or “gold standard” speed (1). These relative speeds are then used to define and prescribetraining intensities, inform crew selection standards, and evaluate performance across specific boat classes (1). This approach has obvious limitations, given that most of the WBTs used to anchor these prognostic speeds are set in the best-case scenarios of athlete performance (i.e., tapered, world class field) and environmental conditions (i.e., tailwind, warm water). Therefore, it is likely that more nuanced adjustments to this method need to be applied in practice.

One such method recently proposed by Kimmins and Tsai (5) involves a further layer of relativity applied within each competitive event in attempt to control for event-specific factors such as the venue, weather conditions, and the strength of the competing field. The winning time of each boat class was reported as a margin from the fastest winning time on that day of competition (most often the Men's Coxed Eight). These margins were calculated from all finals contested at Olympic Games and World Championships between 2005 and 2016 and fed into a linear regression model to generate adjusted prognostic times [see Kimmins and Tsai (5) for methods]. One key assumption in this approach is the stability of weather conditions within a 2 h window in which these events are usually all held. However, field-based observations suggest this is not always the case, with acute variations in weather possible both between and within races, and variable effects of the same weather conditions across different boats in a race depending on lane draw (i.e., wind shadow with crosswind) (2, 8). Regardless, the modelled times performed well in retrospective analysis, correctly forecasting a standard that would produce winning times in 80% (range: 58–100%) of races in the historical data pool (2005–2016). However, when these times were applied to the recent Tokyo 2021 Olympic Games (Table 1), they only accounted for 36% (5/14) of winning times. This is not to say that the fastest crews, as referenced by this adjusted prognostic standard, may not have still been the fastest crews in the favourable conditions of Tokyo; however, it does highlight a limitation in the prognostic speed approach to set performance standards.

Outside of use in selection standards, prognostic speeds are also routinely used to define training intensities and to report athlete progress across a range of specific training efforts (1). Given the high training demands of rowing athletes (9), it is common to assess maximal speed across non-race specific distances and strokes rates (4). Specifically, maximal efforts at sub-race distances or prolonged efforts at submaximal stroke rates are commonly used as proxy measures of performance at various stages of the season. Given the well-established relationship between stroke rate and boat speed (1, 3, 10–12), submaximal stroke rates are often assigned to a scaled prognostic boat speed target. One example of this in the Australian high-performance system is the goal to hit 80% prognostic speed at 20 spm (13). While this method of scaling allows greater access to performance assessment in training, more variable environmental conditions are often encountered in a training vs. competition environment (i.e., water flow, non-buoyed course) which may make interpretation of results difficult. Targeted training sessions at a more controlled venue (i.e., buoyed course), or a session design that involves repeated efforts with and against the prevailing conditions (i.e., into the wind/flow, against the wind/flow) are some methods that may help to mitigate any additional sources of variability.Like the approach of Kimmins and Tsai (5), coaches and practitioners often look to relative performance to scale athletes within each individual session. This also helps give context to the environmental conditions; however, an obvious limitation is the quality of athlete in the training environment (in addition to the reliability of their performances).

Overall, while relative time may offer improved context to results within a given event (i.e., day of competition or single training session), it offers limited scope for assessing change between venues or over time without a greater understanding of the influence from other contributing factors such as the environmental conditions.

## Environmental conditions

The ability to accurately account for environmental influence in on-water performance is of particular interest and importance in rowing. However, the complexity of this task cannot be understated given the dynamic influence of environmental conditions both above (i.e., wind and wave state) and below (i.e., temperature, flow, and depth) the water, plus the downstream influence of these conditions on boat stability and rowers' technique.

The first challenge to overcome in solving this problem is the accurate quantification of all environmental inputs. While there are some basic standards to international rowing courses (i.e., water depth and minimal flow), the other inputs remain unchecked (i.e., wind, water temperature), and this variability is amplified in less-controlled training environments (i.e., variable direction of travel, water flow). Currently, there is no standardised method for collecting and reporting environmental data from international competition, and it is likely that most training environments utilise bespoke systems. This may include single point weather stations near the venue, or the combination of multiple stations placed across a given area to better account for the site variability that may exist. Differences in the intensity and direction of the wind can vary across crews even within the same race (i.e., wind shadow in lanes closer to bank), so this granularity of data may be important (1, 2, 8). An alternative option is boat-mounted sensors that directly quantify the impact of wind on each boat. One such available system is proposed by Kleshnev (1), however, there is limited peer-reviewed data exploring its validity. Even if a high sensitivity of this environmental data was to be captured, the resultant influence on boat speed is another challenge to decipher.

The primary influence of the environment on boat speed in rowing comes from hydrodynamic resistance (14). Wind tunnel experiments have determined that approximately 13% of hydrodynamic resistance comes from aerodynamic drag above the water surface, of which, 50% is from the oars, 35% the rowers’ body, and 15% the boat and riggers (1, 14). These proportions may increase up to 4 times under headwind conditions, but may also reduce to zero in a sufficient tailwind (1, 14), with athlete technique (i.e., squaring of blade) also impacting aerodynamic drag and boat speed (15). The original experimental data from Filter (14) indicated that head-, cross-, and tail-winds may impact boat sizes differently, also varying for the size/shape of the rower (i.e., male vs. female) (14, 16). It is also possible that variability in the wind speed during a race (i.e., wind gusts) creates more complexity than can be fully accounted for with average wind speed measures alone. Below the waterline, the frictional resistance of water against the boat, in addition to wave generation, contribute to other 80% and 7% of hydrodynamic resistance, respectively (1, 14). The former is dependent on water temperature; as water temperature increases, viscosity and density decrease and frictional resistance is thus reduced (14, 17). 4 established a temperature correction factor for boat speed based on experimental data in the 1970s, although data reported by the Australian Institute of Sport found this correction to underestimate the impact of water temperature (17). Water flow presents an additional challenge in training environments that are on flowing waterways. However, hull-mounted impellers, which measure boat speed relative to water flow, can provide a solution to this problem in isolation; although, they are tedious to setup, calibrate, and are often impacted by river debris.

Despite the challenges of capturing and correcting for the influence of environmental conditions on boat speed, numerous attempts have been made (18–22). One such practical example of how environmental corrections may be applied is shown in Table 2. Displayed here are results released by Rowing Australia from the underage national team trials conducted in Canberra (ACT) in May 2022. Here, crew combinations were raced over 1500 m or 1800 m. Custom weather stations were placed at intervals along either side the course (0, 300, 800, 1300, and 1800 m) to capture wind speed and direction just above the water level. Water temperature was also collected and reported for each race. Table 2 shows repeat race performances from identical crew combinations within each boat class, and average race speeds are corrected to standardised environmental conditions (0 m/s wind and 26°C water), which are chosen to represent ideal conditions in European racing season. Water temperature correction was performed using a regression equation reported by Lazauskas (17). Wind corrections were based on work from Filter (14); however, adjustments were scaled to 50% of those provided based on historical trialling of this model. While the conditions reported here were mild (0–2.4 m/s wind), there did appear to be an improvement in the reliability of performance times for the same crews, compared to the raw times which showed a bias towards the colder water temperatures. Of course, there are obvious limitations with this example, given the variable race distances and relatively small sample, however, it does highlight the potential benefit of environmental correction to provide a more analogous performance standard across different environments. Modelling for the prediction of expected/required performance times at an upcoming competition, or the retrospective analysis of standardised competition results captured across a range of different environmental conditions is of significant value to rowing coaches and practitioners. To build upon some of the existing work in this area (2, 3), more data is needed across a broader range of environmental conditions, and given the complexity of this task, a more explicit and consistent methodology for environmental capture and correction is required. Furthermore, recent work from Holt and colleagues (3) has highlighted the value of including additional contextual measures of rowing such as stroke rate and power to compliment the prediction of on-water performance.

**Table 2:** Repeat race results for the same crew combinations, collected between the 1st and 8th of May 2022 at the same location (Canberra, AUS). Average speed from each race used to project a 2000m race time. Water temperature and wind speed used to calculate a corrected speed and projected 2000m race time for standardised environmental conditions (0 m/s wind and 26° water). Margin for race time differences calculated within each crew and presented as a mean difference with a 99% confidence interval.

Boat Class	Race #	Race Distance (m)	Water Temperature (°C)	Wind Speed (m/s)	Wind Direction	Raw Speed (m/s)	Raw 2000m Time Projection (mm:ss.0)	Raw Margin (sec)	Corrected Speed (m/s)	Corrected 2000m Time Projection (mm:ss.0)	Corrected Margin (sec)
JM4-	1	1500	15.8	0.0	Cross Tail (106°)	5.116	06:30.9	**3**.**71**	**5**.**359**	**06:13.2**	**3**.**60**
	2	1800	14.1	1.1	Cross Head (63°)	5.165	06:27.2		**5**.**308**	**06:16.8**	
JM4+	1	1800	16.0	0.1	Cross Head (33°)	5.085	06:33.3	**−1**.**16**	**5**.**184**	**06:25.8**	**−2**.**00**
	2	1800	14.1	1.0	Cross Head (60°)	5.071	06:34.4		**5**.**211**	**06:23.8**	
JM8+	1	1800	16.4	0.2	Cross Head (79°)	5.662	05:53.2	**−4**.**61**	**5**.**767**	**05:46.8**	**1**.**44**
	2	1800	14.1	1.2	Cross Head (61°)	5.589	05:57.8		**5**.**743**	**05:48.2**	
JW2x	1	1800	16.2	0.3	Cross Head (59°)	4.418	07:32.7	**−6**.**32**	**4**.**500**	**07:24.4**	**2**.**22**
	2	1800	14.1	1.4	Cross Head (57°)	4.357	07:39.0		**4**.**478**	**07:26.7**	
JW8+	1	1500	15.8	0.0	Cross Tail (106°)	5.002	06:39.9	**−6**.**87**	**5**.**112**	**06:31.2**	**4**.**58**
	2	1800	14.1	1.1	Cross Head (60°)	4.917	06:46.7		**5**.**053**	**06:35.8**	
U23LW1x	1	1800	16.0	0.8	Cross Head (50°)	4.418	07:32.7	**−6**.**32**	**4**.**151**	**08:01.8**	**−2**.**44**
	2	1800	14.1	1.2	Cross Head (57°)	4.357	07:39.0		**4**.**172**	**07:59.3**	
U23LW2-	1	1800	16.0	1.0	Cross Head (44°)	4.129	08:04.4	**2**.**56**	**4**.**230**	**07:52.8**	**−3**.**89**
	2	1800	14.1	1.3	Cross Head (57°)	4.151	08:01.8		**4**.**265**	**07:48.9**	
U23M1x	1	1800	16.0	1.0	Cross Head (44°)	4.605	07:14.3	**0**.**87**	**4**.**714**	**07:04.3**	**−2**.**53**
	2	1800	14.1	1.4	Cross Head (57°)	4.614	07:13.4		**4**.**742**	**07:01.8**	
U23M4-	1	1500	16.4	2.4	Cross Tail (152°)	5.517	06:02.5	**−11**.**18**	**5**.**562**	**05:59.6**	**3**.**96**
	2	1800	14.1	1.2	Cross Head (62°)	5.352	06:13.7		**5**.**501**	**06:03.6**	
U23M4+	1	1500	16.8	2.1	Cross Head (291°)	5.193	06:25.1	**−2**.**94**	**5**.**287**	**06:18.3**	**−0**.**60**
	2	1800	14.1	1.1	Cross Head (62°)	5.154	06:28.1		**5**.**296**	**06:17.7**	
U23M4x	1	1500	16.4	0.8	Cross Head (323°)	5.423	06:08.8	**0**.**96**	**5**.**547**	**06:00.5**	**−2**.**64**
	2	1800	14.1	1.2	Cross Head (61°)	5.438	06:07.8		**5**.**588**	**05:57.9**	
U23W1x	1	1800	16.0	1.0	Cross Head (58°)	4.168	07:59.9	**0**.**09**	**4**.**267**	**07:48.7**	**−1**.**78**
	2	1800	14.1	1.3	Cross Head (57°)	4.168	07:59.8		**4**.**284**	**07:46.9**	
**Average Margin (sec)**								**−2.60**			**−0**.**01**
**99% Confidence Interval (sec)**								**(−5.96–0.75)**			**(−2.23–2.22)**

M, Men's; W, Women's; L, lightweight; J, junior/U19 age category; U23, U23 age category; 1x, single sculls; 2x, double sculls; 2-, Pair; 4x, quadruple sculls; 4-, four; 4+, coxed four; 8+, eight Wind direction represented as relative direction of boat travel; 0°, direct head; 90°, cross from bow side; 180°, direct tail, and 270° = cross from stroke side.

## Power instrumentation

One potential avenue for developing a greater understanding of any given boat speed or race time in rowing is the assessment of kinetic data made available through the instrumentation of boats (2). In water-based sports, there is a theoretical curvilinear relationship between power and speed (3, 23, 24), with every 1% increase in boat speed requiring a 2.9–3.7% increase in power output to overcome water resistance (3). While this relationship can be impacted by environmental conditions and athlete characteristics (i.e., body weight, technical efficiency), measures of on-water power may ultimately provide a more direct assessment of exercise intensity and boat performance (10, 24, 25). To evidence this, Holt and colleagues (3) found that mean power output during a 2000 m on-water race had the largest modifying effect on the prediction of race speed, when also accounting for measures of stroke rate and head wind. Knowledge advancement in this area has clear value for coaches and practitioners, with the potential to help guide preparation towards desired race speeds using target powers in the training environment. As previously raised, consideration must be given to the added variable of water flow in some training environments and the impact this may have on the power vs. speed relationship. Accordingly, work from Hogan and colleagues (24) in the sport of sprint kayak demonstrated a de-coupling of the power vs. speed relationship when working at a set intensity (power and stroke rate) up and down a flowing river. This was corrected for when power was compared to speed measured *via* a boat mounted impeller, as this better accounts for movement speed direct to the water when compared to traditional land-based point to point speed (i.e., GPS speed). Accordingly, rather than trying to individually account for all the external environmental factors that may influence boat speed (i.e., wind and flow), power may provide a more holistic and robust method of performance assessment in on-water rowing. Further, power may provide additional benefit in the training setting as an instantaneous feedback tool, with evidence to show a 65% improvement in training intensity adherence compared to coach feedback, boat speed, and stroke rate alone (25).

When compared to more technologically advanced sports such as cycling, it is evident that the assessment of on-water power in rowing has widespread potential for the quantification of performance through relation to power-based speed and physiological benchmarks (26). As such, the concurrent validity of some of the available instrumentation systems was examined, with the Peach system (Peach Innovations, Cambridge, United Kingdom) recommended as the most valid and reliable of those tested (27). However, it should be noted that there is a significant cost (approximately ∼$1000–$2000 AUD/seat) associated with these oarlock devices, as well as an additional weight burden in the boat (approximately 250–400 g/seat; cost and weight estimates from Peach Innovations Ltd., http://www.peachinnovations.com/**)**. There have also been some conceptual discussions raised about the proxy measure of power output provided by the commonly used oarlock instrumentation systems; however, since this method does not account for all forces acting on the boat and athlete system (i.e., foot stretcher force and athlete acceleration), it has been suggested to underestimate true mechanical power by approximately 10% (3, 28, 29). Therefore, Lintmeijer and colleagues (28) suggested that in cases where calculation of a true mechanical power output is required, such as when comparing on-water to lab-based ergometer training, or when prescribing training intensity using instantaneous power-output feedback, a correction factor should be applied to the proxy measure. Further, it must be acknowledged that power output measures alone should not replace a more comprehensive assessment of rowing performance, and additional variables including stroke rate and technical efficiency should be considered wherever possible to better explain both individual athlete and boat performance (3).With acknowledgement of these limitations, measures of on-water power output have great potential for use in furthering coach, practitioner and athlete understanding of the performance standard in on-water rowing.

## Conclusion

This paper discusses the current challenges associated with capturing a true performance in on-water rowing, with raw boat speeds and race times influenced by a myriad of factors in both competition and training environments. Relative time comparisons may provide the most accessible method of adding context to results within a given event (i.e., day of competition or single training session), but has limited scope to assess longitudinal change or perform comparisons between venues. Quantification of the environmental influence is extremely complex, however, there does appear to be some merit in correcting for water temperature and wind speed/direction in mild conditions. More work should be directed towards setting a standard for environmental data collection and the validity of environmental corrections across a broader range of conditions and boat classes. Power measurement is a promising new avenue for exploration, with the potential to add more contextual understanding to both individual athlete and boat performance in an on-water setting. More research is encouraged to further develop our understanding of the power vs. speed relationship, uncover world class performance standards for these new available measures, and challenge the validity of information transfer between training and competition environments.

## Data Availability

The original contributions presented in the study are included in the article/Supplementary Material, further inquiries can be directed to the corresponding author/s.
